# Reclaiming the primary care consultation for patients and clinicians: is AI-enabled ambient voice technology the answer?

**DOI:** 10.1177/01410768251360853

**Published:** 2025-07-17

**Authors:** Ahmed Alboksmaty, BWJ Hayhoe, A Majeed, Ana-Luisa Neves

**Affiliations:** 1 Institute of Global Health Innovation, Department of Surgery and Cancer, Imperial College London, London SW7 2AZ, UK; 2 Department of Primary Care and Public Health, School of Public Health, Imperial College London, London W120BZ, UK

Electronic health records (EHRs) are now ubiquitous in primary care in most developed countries, offering significant benefits for patient safety and care quality.^1^ However, as their functionality and complexity have increased, they increasingly divert clinicians’ attention from patients.^2^ This is particularly true in systems like the National Health Service (NHS) in the UK, where the funding model makes EHR use essential for the survival of primary care practices.^3^ More broadly, the digitalisation of primary care and growing reliance on electronic documentation, coding and data entry to meet administrative, financial and regulatory requirements have added complexity to clinical workflows.^4^ These digital demands can disrupt traditional doctor–patient communication, making it harder for general practitioners (GPs) to maintain direct, person-centred interactions.^2,5^

Clinicians can spend twice as much time on EHR tasks, such as documentation and order entry, as on direct patient care, creating a sense of administrative burden.^2^ During consultations, focusing on EHRs for documentation can leave both patients and clinicians feeling that the interaction is deprioritised.^2,5–7^ Patients value being the focus of their clinician’s attention, yet computer use can make clinicians appear distracted, less compassionate and less professional.^5,8,9^ Clinicians report administrative burden, burnout risk and misalignment with patient-centred care in these situations.^2,4^ Moreover, non-verbal cues may be overlooked, potentially compromising patient safety.^10,11^

## Can technology reverse its own disruptive effects to enhance patient-centred care?

The recent independent NHS review emphasised that digital transformation is still evolving, urging wider adoption of artificial intelligence (AI)-driven tools to enhance care quality and safety.^12^ AI-enabled ambient voice technology (AVT) is one such tool and is strongly promoted by healthcare authorities and policymakers.^13,14^ Through real-time transcription of patient–clinician conversations into structured summaries that can be potentially entered automatically into the medical record, AVT offers a possible solution to the disruptive effect of EHRs in consultations, while improving documentation quality and information sharing.^15^ AVT can help clinicians maintain focus on patients and their care, enhancing communication, patient experience and overall care quality in primary care and beyond.^15,16^

Furthermore, as of November 2023, all NHS GP practices are contractually required to provide patients with online access to newly added information in their records, including consultation notes.^17^ This NHS guidance has been informed by previous research linking record access to improved health outcomes.^18^ However, for records to be useful to patients, they must be high quality, detailed, clear and easily understandable (i.e. avoiding jargon).^18^ AVT may support clinicians in achieving this by enabling comprehensive, patient-friendly documentation and generating post-consultation summaries.^19^ It may also improve communication between primary and secondary care through generation of specialist referral letters when needed.^20^ This aligns with the NHS Long Term Plan and Department of Health and Social Care priorities promoting AI-powered tools in delivering accessible, digitally-enabled care.^21^

## Does evidence support the use of AVT in clinical consultations?

Preliminary research has explored the impact of AVT, showing its potential to improve care quality through this evolving technology.^15,16,22^ A systematic review by Falcetta et al. identified AVT as a promising tool for improving consultation efficiency and reducing documentation time while maintaining accuracy.^22^ A more recent review by our team (covering studies up to September 2024) further highlighted AVT’s role in enhancing primary care quality, particularly in effectiveness, efficiency and patient-centredness. Furthermore, a government-led initiative across London evaluated AVT implementation in multiple sites and diverse healthcare settings, including primary care, and reported improvements in clinical productivity and the quality of patient interactions during consultations.^14^

While current evidence suggests several benefits of AVT, some concerns remain. First, data security remains a critical concern, particularly how sensitive patient data are processed, stored and shared.^13,23^ Second, AVT accuracy varies, yet all AVT systems may introduce errors into clinical summaries,^24,25^ which may embed misinterpretations into permanent records. Finally, AVT may reshape consultations, with clinicians potentially adjusting communication to suit the AI rather than the patient.^15^ It is important to note that NHS England has, in recently published guidance on implementation of AVT, indicated that ultimate accountability remains with the healthcare providers, recommending that organisations establish clear contractual agreements with AVT suppliers that delineate responsibilities, obligations and liability arrangements.^13^

While interest in AVT, particularly in primary care, continues to grow, evidence regarding its feasibility, acceptability and real-world impact in primary care remains limited. This includes data on cost, staff training and implementation (including integration with current electronic medical record systems). Questions also remain about how AVT handles complex consultations, including with non-native English speakers, and its broader integration into routine practice. As AVT develops, further research is needed to assess its usability, acceptability, feasibility, cost-effectiveness and unintended effects on clinical interactions and decision-making.^26^ If demonstrated to be effective, AVT could become a transformative tool in advancing NHS Long Term Plan objectives.^21^

## Declarations

### Guarantor

AA, BWJH, AM, ALN.

### Provenance

Not commissioned; editorial review.
